# Structural, programmatic, and sociocultural intersectionality of gender influencing access-uptake of reproductive, maternal, and child health services in developing regions of Ethiopia: A qualitative study

**DOI:** 10.1371/journal.pone.0282711

**Published:** 2023-03-07

**Authors:** Yohannes Kebede, Firanbon Teshome, Wakgari Binu, Ayantu Kebede, Anwar Seid, Habtamu K. Kasaye, Yibeltal Kiflie Alemayehu, Wondimagegn Tekalign, Girmay Medhin, Yared Abera, Derebe Tadesse, Mulusew J. Gerbaba

**Affiliations:** 1 Faculty of Public Health, Department of Health, Behavior and Society, Jimma University, Jimma, Ethiopia; 2 School of Public Health, Wolaita Sodo University, Wolaita Sodo, Ethiopia; 3 Faculty of Public Health, Department of Epidemiology, Jimma University, Jimma, Ethiopia; 4 Department of Nursing, College of Medical and Health Sciences, Samara University, Semera, Ethiopia; 5 Institute of Health Science, Wollega University, Nekemte, Ethiopia; 6 Faculty of Public Health, Department of Health policy and management, Jimma University, Jimma, Ethiopia; 7 MERQ Consultancy PLC, Jimma, Ethiopia; 8 IntraHealth International Ethiopia, Addis Ababa, Ethiopia; 9 Aklilu Lemma Institute of Pathobiology, Addis Ababa University, Addis Ababa, Ethiopia; 10 USAID Transform Health in Developing Regions, Amref Health Africa in Ethiopia, Addis Ababa, Ethiopia; Chinese Academy of Medical Sciences and Peking Union Medical College, CHINA

## Abstract

**Background:**

Gender remains a critical social factor in reproductive, maternal, and child health and family planning (RMNCH/FP) care. However, its intersectionality with other social determinants of the RMNCH remains poorly documented. This study aimed to explore the influence of gender intersectionality on the access uptake of RMNCH/FP in Developing Regional States (DRS) in Ethiopia.

**Methods:**

We conducted a qualitative study to explore the intersectionality of gender with other social and structural factors and its influence on RMNCH/FP use in 20 selected districts in four DRS of Ethiopia. We conducted 20 Focus Group Discussions (FGDs) and 32 in-depth and key informant interviews (IDIs/KIIs) among men and women of reproductive age who were purposively selected from communities and organizations in different settings. Audio-recorded data were transcribed verbatim and analyzed thematically.

**Findings:**

Women in the DRS were responsible for the children and families’ health care and information, and household chores, whereas men mainly engaged in income generation, decision making, and resource control. Women who were overburdened with household chores were not involved in decision-making, and resource control was less likely to incur transport expenses and use RMNCH/FP services. FP was less utilized than antenatal, child, and delivery services in the DRS,as it was mainly affected by the sociocultural, structural, and programmatic intersectionality of gender. The women-focused RMNCH/FP education initiatives that followed the deployment of female frontline health extension workers (HEWs) created a high demand for FP among women. Nonetheless, the unmet need for FP worsened as a result of the RMNCH/FP initiatives that strategically marginalized men, who often have resource control and decision-making virtues that emanate from the sociocultural, religious, and structural positions they assumed.

**Conclusions:**

Structural, sociocultural, religious, and programmatic intersectionality of gender shaped access to and use of RMNCH/FP services. Men’s dominance in resource control and decision-making in sociocultural-religious affairs intersected with their poor engagement in health empowerment initiatives that mainly engaged women set the key barrier to RMNCH/FP uptake. Improved access to and uptake of RMNCH would best result from gender-responsive strategies established through a systemic understanding of intersectional gender inequalities and through increased participation of men in RMNCH programs in the DRS of Ethiopia.

## 1. Background

Reproductive, Maternal, Newborn, Child Health, and Family Planning (RMNCH/FP) services encompass newborns, children, and women’s healthcare before, during, and after pregnancy [1]. Equitable, accessible, safe, and quality RMNCH/FP care are the key interventions to improve maternal and child care services and achieve sustainable development goals (SDG) 3 (health and well-being) targets 3.1, 3.2, and 3.7 [2–6]. However, maternal and child morbidities and mortalities continue to be major public health problems in low- and middle-income countries (LMICs) [7–10].

Research from LMICs has shown that gender inequities, sociocultural beliefs and perceptions, policies, and program and service delivery approaches influence RMNCH/FP service access and utilization [1–3,7–10]. Gender inequalities could lead to unequal participation in households, social and health activities, and leadership [11–13] and contribute to adverse health and RMNCH outcomes in multiple pathways [14–19]. For instance, restrictive gender roles and inequalities negatively impact the health of women and children by limiting women’s capacity by influencing decision-making power regarding age at marriage, birth spacing, and limited access to and control over resources (e.g., finances, information, transport, and supplies) [12,13,17–23]. The role of gender in antenatal, delivery, and postnatal care utilization has also been demonstrated in various countries [15,24–30] such as Uganda [15], Kenya [25], and Ethiopia [26,27]. Studies from Nepal [28], Cameroon [29], and Nepal [30]. Gender intersects with other social determinants of health such as ethnicity, religion, culture, social class, and economy, exacerbating adverse health outcomes among women and children [31,32]. For example, women who are poor and belong to disadvantaged ethnicities have lower access to healthcare than those who are poor but belong to advantaged ethnic groups [25]. Lack of “female centered” services and culturally competent skills from the health systems side have a compounded effect on maternal health care services utilization [20].

Although efforts have been made to close gender disparities in Ethiopia [33–35], gender inequalities are still widely observed [36–42]. This study aimed to explore the intersectionality of gender with other social factors that influence access and uptake of RMNCH services. Understanding the extent to which gender intersects with other social stratifiers and programmatic factors would have policy and operational relevance in maternal and child health services. Specifically, we report 1) gender norms and their influence on the uptake of RMNCH, and 2) the intersectionality of gender with structural, sociocultural, and programmatic contexts (decision-making, resource management, program/service arrangements and delivery, and sociocultural and psychological practices) in influencing access to and uptake of RMNCH services.

## 2. Methods

### 2.1 Study contexts

There is differential heterogeneity in overall health development within and between Ethiopia’s regions. To narrow this gap, the government of Ethiopia, in collaboration with development partners, has been implementing various interventions in Afar, Benishangul-Gumuz, Gambella, and Somali the least developing region of Ethiopia under the Transform Health in Developing Regions (HDR) project. The DRSs are predominantly pastoral (characterized by limited access to information, weak health systems, limited availability of health facilities and health staff, and seasonal mobility), accounting for 52% of the country’s landmass, and have relatively low RMNCH service utilization compared to the agrarian regions and national averages. For instance, according to the 2016 Ethiopian Demographic Health Survey (EDHS), the contraceptive prevalence rate (CPR) in the Afar and Somali regions was 12% and 1%, with the highest total fertility rates of 5.5 and 7.2 children per woman, respectively. Institutional delivery assisted by skilled attendants was comparatively low: 16.4% for Afar, 20% for Somali, 28.6% for Benishangul-Gumuz, and 46.9% for the Gambella regional states. These regions also have the highest under-five mortality rate, far from the national average of 67 deaths per 1000 live births: 125 in Afar, 94 in Somali, 98 in Benishangul-Gumuz, and 88 in Gambella [43]. Ethiopia ranked 97^th^ of 157 countries globally in the gender gap index estimated at 0.69 in 2021[44]. The USAID Transform HDR project aims to attain 50% of the health sector transformation plan’s RMNCH-related indicators by 2022 by improving existing government efforts and strengthening health systems by increasing access to integrated, quality, and high-impact RMNCH/FP services, improving health-seeking behaviors by reducing gender inequalities, and improving evidence-based decision-making and program learning.

### 2.2 Study area and period

The study was conducted from March 3 to 26, 2019, in 40 kebeles (the lowest government administrative unit) selected from 20 districts nested under 20 zones of four developing regional states (DRS).

### 2.3 Study design

We conducted an exploratory qualitative study to assess the gender intersectionality of sociocultural, structural, and programmatic barriers and facilitators of RMNCH/FP service access and use. The focus was on an in-depth understanding and interpretation of the settings and people’s feelings, experiences, perceptions, choices, and preferences regarding gender and other social factors of RMNCH/FP. The gender analysis framework for health systems was used to elicit concepts regarding how gender norms interact with the structural, sociocultural, and programmatic contexts associated with RMNCH/FP services.

### 2.4 Participants and sampling

First, we selected 20 zones from the four regions and chose one district from each zone and two kebeles/villages from each district, for a total of 40 kebeles. Purposive sampling was used to select study settings and participants. Districts and kebeles selection was guided by RMNCH/FP service performance and coverage rates in consultation with DRS health offices, whereas participants were selected by referring to RMNCH/FP use/non-use status, sex, and age groups from the community and health facilities. The study participants included men and women (including boys and girls) of reproductive age (15–49 years of age), representatives of regional, zonal, district, and kebele-level government offices (health, women and children affairs, labor and social affairs, and health facilities), and religious and community leaders.

### 2.5 Data sources and respondents

We conducted key informant interviews (KIIs), in-depth interviews (IDIs), and focus group discussions (FGDs) using **S1 File**. The sample size included 20 FGDs and 32 IDIs/KIIs, resulting in 52 interviews. KIIs were collected from regional offices, NGO partner staff, HEWs, Health Center staff, district health offices, district women and children affairs offices, district labor and social affairs offices, and religious and clan leaders. IDIs were performed with RMNCH service users and nonusers. Age, sex, and RMNCH user status were considered in the FGDs (Table 1). On average, there–8–12 individuals participated in the FGDs. Diversity of data was ensured as IDIs and FGDs were with men and women, with two age groups (young:15–24, and adult:25–49), RMNCH service use status (ANC, FP, and child health users/nonusers), and regional/zonal/district/kebele representations.

**Table 1:** Data sources by regions, gender intersectionality and MCH study, DRS of Ethiopia, 2019.

Selected number of settings			Data sources	Total
**Regions**	Districts	Kebele		
**Afar**	5	10	FGDs	5 FGDs
			IDIS	4 IDIs
			KIIS	4 KIIs
**Somali**	5	10	FGDs	5 FGDs
			IDIS	4 DIs
			KIIS	4 KIIs
**Gambella**	5	10	FGDs	5 FGDs
			IDIS	4 IDIs
			KIIS	4 KIIs
**Benishangul-Gumuz**	5	10	FGDs	5 FGDs
			IDIS	4 IDIs
			KIIS	4 KIIs
**Total**	20	40	-	52 sample

### 2.6 Data collectors and field data collection

Fifteen interviewers (one female and 14 males) with a master’s degree and experience in qualitative data collection conducted the KIIs, IDIs, and FGDs. They received two-day training on the study objective, tools, ethical issues, and sampling criteria. Participants’ selection and arrangements for the interviews and discussions were assisted by local guiders: Health Extension Workers (HEWs) and women’s development army (WDA) leaders. All interviews were audio recorded. Notes were taken while in the field. Data collection was supervised by daily debriefings and discussions on idea saturation, field experience, general impressions, and challenges. Data collection and analysis were performed iteratively.

### 2.7 Data analysis

First, we transcribed the record verbatim, translated it into English, and uploaded it to NVivo software for coding **S2 File**. A thematic analysis using open coding was applied. Field notes were used to support the coding and interpretation. Before open coding, three experienced data coders independently read and re-read each transcribed document to identify the rich text data obtained from the FGDs, IDIs, and KIIs to generate the initial codebook. Accordingly, we identified ten text-rich data to initiate the independent coding process, then discussed the initial codes and reached a consensus on the code definitions, after which we added the newly emerged codes to develop a refined code. We read, re-read, and clustered the codes to develop the categories, subthemes, and main themes. We provided thick descriptions of the contexts and triangulated the data to substantiate the interpretations of sub-themes and categories. Finally, we presented the results in themes, sub-themes, and categories, with quotations supporting the underlying concepts.

### 2.8 Rigor

The credibility of our findings was confirmed in this study. The data were collected and managed by experienced qualitative researchers with PhD and master’s degrees and knowledge of the health system, gender, and RMNCH. Nonetheless, they bracketed themselves from intentionally providing expertise and reflexive meanings with minimal interpretation bias. Supervisors closely monitored the quality of transcriptions against actual audio recordings. Tick descriptions of the data and themes were presented with supportive quotations that added value to credibility. Subjective neutrality, peer debriefing, daily interactions with the research team, and audit trials were considered to ensure the credibility and dependability of the findings. The diversity and triangulation of data (FGDs, KII, and IDIs) by region, district, kebele, age, gender, and RMNCH use status can enhance the transferability of the findings to DRS and similar contexts. Additionally, the saturation of ideas in the data, richness, and credibility of the findings are confirmed through empirical and theoretical evidence on gender and its sociocultural intersectional influences on the health system, RMNCH, and socio-ecological perspectives.

### 2.9 Ethics statement

Ethical clearance was obtained from the Ethiopian Public Health Institute (EPHI-IRB-143-2018). Verbal informed consent was obtained from participants after providing information on the objectives of the assessment and the benefits/risks associated with their participation. The participants understood the purpose of the study and the topics they discussed during the interviews. In addition, the collected information was anonymized, as personal identification information was not reported. The interviews were kept confidential and private, as they were conducted in private rooms and spaces in the community. The audio records and transcriptions were stored on a private computer. In addition to informing the purpose of the study and how the findings inform RMNCH/FFP services, the interviewers maintained prolonged engagement with all participants to facilitate interviewees’ free and objective responses in a non-judgmental manner.

## 3. Findings

The key themes that emerged on gender interaction and intersectionality with structural, programmatic, and sociocultural dimensions to influence RMNCH/FP service access uptake in the DRS of Ethiopia were gender norms in RMNCH; and structural, sociocultural, and programmatic conditions intersecting gender norms and access and use of RMNCH. The main themes and sub-themes are listed in Table 2.

**Table 2:** Definitions and descriptions of themes and sub-themes of intersectionality of gender and sociocultural factors of RMNCH, March 2019.

Sn	Themes and sub-themes	Definitions and descriptions
**1**	Gender norms and roles in RMNCH a- Livelihood/productive roles and norms b- Reproductive roles/norms	Livelihood and RMNCH-related roles (expectations) and norms (shared roles at varying intensity) played by men and women in developing regions
	Gender norms, shifts, and uptake of RMNCH	How the gender norms shaped access-use of RMNCH services
	Cross-intersectionality of gender and RMNCH	Cross interactions of Structural, cultural, program, and gender and RMNCH
**3.1.**	Structural intersectionality of gender and RMNCH a-Decision making and leadership b- Power over the control of resources c- Laws and policies d- Empowerment opportunity	This theme broadly explains formal structures such as policies and laws, leadership and power, empowerment opportunities or challenges underlying access-use of RMNCH services, and interacting gender norms
**3.2**	Programmatic intersectionality of gender and RMNCH a- Access to RMNCH services b- Preferences for health workers and services	This construct discussed RMNCH program structure and organization interacted and adapted to gender contexts in influencing the access-uptake of RMNCH services
**3.3**	Socio- cultural, psychological and demographic intersectionality of gender and RMNCH a- Socio-demographic b- Sociocultural -religious practices c- Knowledge, perceptions, and beliefs	By this theme, the study explained how sociocultural, livelihood, and psychological factors intersected with gender norms and access-use to RMNCH

**Fig 1:**
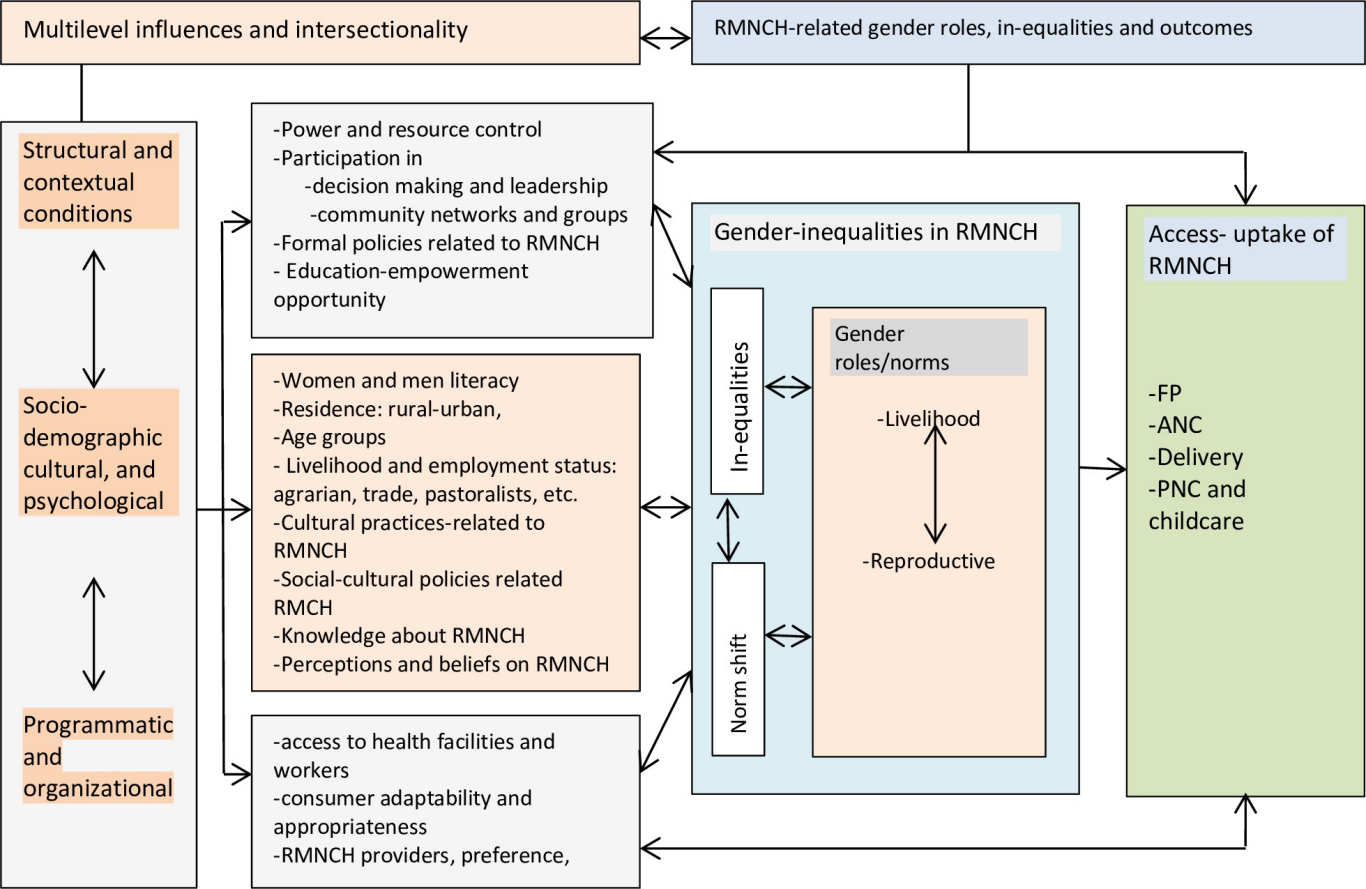
Diagram indicating gender intersectionality in shaping access-use of RMNCH developing regions of Ethiopia Note: Reciprocal arrows indicated intersectionality across barriers-facilitators conditions of access-use of RMNCH.

As indicated in Table 2, gender influenced access to and use of RMNCH services in the DRS through multilevel and intersectional factors. Fig 1 visualized the gender intersectionality of these factors and how they shaped RMNCH service use. Fig 1: Diagram of the findings.

### 3.1. Gender roles, norms, and inequalities of engagement in RMNCH

Gender roles (tasks assigned to either men or women) were immediate factors for access to RMNCH services in the DRS of Ethiopia. We referred to tasks shared by both men and women, but at unequal intensities, as norms. In this study, the theme “gender norms and roles” were broadly grouped into two interdependent sub-themes: livelihood/productive and reproductive roles. Productive roles include routine task sharing and autonomy, such as income-generating activities, partner support, engaging in social affairs, and decision-making. Income generation activities, such as farming/mining, partner support, and engagement on social occasions, are some examples of gender norms. Reproductive roles embraced caring for children and their families, seeking RMNCH services and providers, and seeking RMNCH information.

#### 3.1.1. Gender and productive roles

*Routine task sharing*: *household chores and livelihood*. The majority of the study participants mentioned that the community predominately assigned tasks, such as grain grinding, collecting firewood, preparing food for the family, fetching water, cleaning household utensils, dismantling small huts, and animal husbandry activities by gender. In all regions, men were excluded from childcare and household activities, although they often engaged in income generation. In contrast, women were overburdened with household chores and had limited roles in generating revenue.

*“…. while men often engage in income generation, farming lands, and trades, women often work in house cooking foods and caring children…” (FGD, women from Somali)*

*Women autonomy and power relations*. This study revealed that women were less powerful in easily going out of home to seek health care, participated in income-generating activities and social-cultural occasions, and had limited power over resources. For instance, women demand their husbands’ approval of to visit health facilities for FP use. Women need to present acceptable reasons other than FP to convince their husbands and endorse their visit.

*“…in those households in which the man is dominant, then he can sell the animals and have the money for himself.” (KII, religious leader, Bambasi District)*

*“…. The men don’t allow the women to go far out of home and engage on trades and generate income… and they even want to endorse their visit to health facilities” (FGD, women from Somali)*

#### 3.1.2. Gender and reproductive roles

These roles consider the behavior of caring for and maintaining the health of families and children, as well as the uptake of family planning, pregnancies, and childbirth services.

*Taking care of children*. The majority of the study participants pointed out that taking care of their children was their mother’s duty and responsibility.

*“Women are responsible for taking care of their children. It is solely the responsibility of the woman. Man’s role is minimal regarding taking care of children.” (KII, LSA, Dangur District)*

*Seeking RMNCH services and providers*. First, men dominantly decide on the agenda of FP services, whereas women can decide on ANC and child immunization services.

*“Regarding decision making on the use of RMNCH services: women alone can decide, but in case of family planning, she must discuss with her husband for approval.” (KII, WCA, Lare District)*

*“…..Other than this, it is the woman who in the majority of the time, takes children for vaccination or follows her antenatal or postnatal follow-up alone. But during delivery or labor, it is culturally mandatory for the husband to attend to her ….It is not out of rudeness for the men not to be helpful on the other RMNCH/FP services, it is however originating from their cultural practices.….” (WCY Affairs Office, Gulina District)*

Surprisingly, although men had reservations about FP, they would compromise and permit FP use on certain occasions:1) Women’s past delivery was through cesarean section. In this case, FP was perceived as helpful in delaying pregnancy, potentially leading to illness, and 2) the wives had health complications arising from previous childbirth or medical issues. Unmarried young people were not allowed to use FP services.

*“…Generally, FP is considered as ‘haram’ unless it’s indicated after surgical and medical problems that warn the women not to get pregnant. Unless and otherwise, they do have a positive attitude towards other reproductive services…” [Female FGD, Afar region]*

*“…young and unmarried people are never permitted to use FP service. They are condemned and outcaste by religious and clan leaders if they are found while using FP and other service such as abortion care as getting pregnant is unthinkable among young ladies. “[Females, young FGD,Somali region]*

*Seeking RMNCH information*. Associated with the responsibility that women have to maintain the health of children and families and ensure that the sick are treated, they are supposed to seek even more accessible RMNCH information than men do. Women are targeted by RMNCH/FP education, such as health talks with HEWs and discussions with women/health development armies (HDA).

*“…Females have better access to education than males because females have a better chance to meet the HEWs as they are more responsible for families and children’s health and to be targeted by the school clubs for RH awareness-raising initiatives in the school.” (KII, health center head, Lare District in Afar)*

Nonetheless, young females are often excluded from RMNCH/FP education, given that such matters are not openly discussed at a young age or among unmarried ones.

*“We do not have access to any community based education about sexual matters and the related services as discussing such matters between parent, health workers, and young adolescent like us is considered taboo. We only access information related to sexual health and FP through school or mass media…” (Female, young FGD, Afar region]*

### 3.2. Gender norms/roles on uptake of RMNCH services

**Fig 2:**
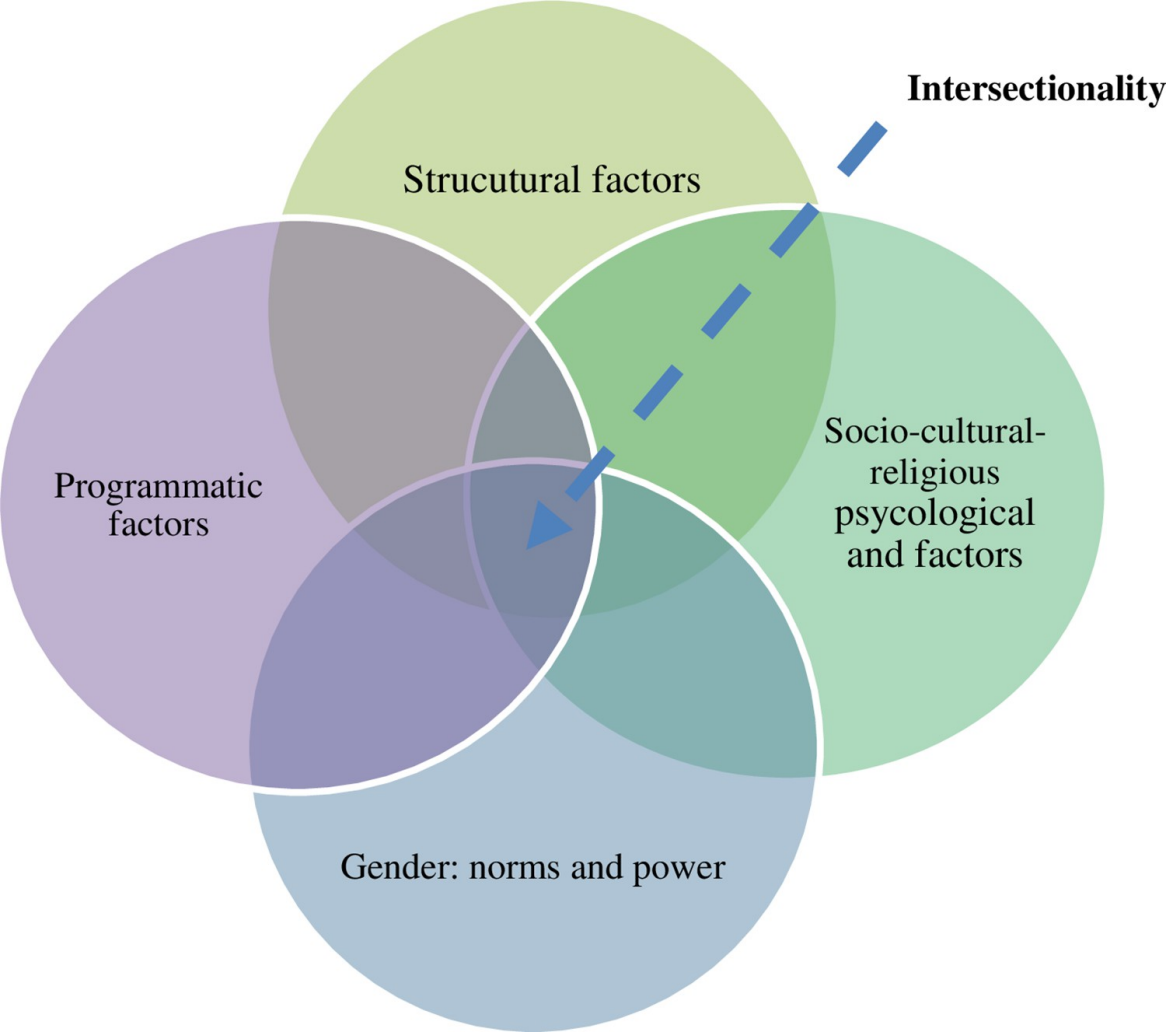
Multi-level intersectionality of structural, socio-cultural, programmatic factors and gender in RMNCH.

As demonstrated in Fig 2, productive and reproductive gender roles appeared to be immediate factors affecting access and useof RMNCH services while distally intersecting and interacting with multilevel structural, cultural, and programmatic conditions. Fig 2: Intersectionality of gender.

*Overburden with roles influence RMNCH uptake*. The assignment of women to more productive roles negatively influences the proper execution of reproductive roles and creates a barrier to RMNCH uptake. Owing to the high burden of household chores, women missed their follow-up dates for ANC, FP services, delivery, and postnatal care/schedules.

*“We do not have any spare time, and we are more burdened with our household activities than men. There is a challenge to go to the HC anytime we want due to the burden we have with our household chores; it is difficult to use the services as needed (Female youth, FGD, Telalak District)*.

Interestingly, some women indicated that neither being overburdened with household chores nor lack of time could be a reason for not seeking RMNCH services, as they felt personal agency to control them.

*“I spend most of my time performing household duties, such as preparing food for the family, washing household utensils, and taking care of my children. These household chores do not influence my health-seeking behavior and access to RMNCH services. I can go to a health facility and seek medical care and treatment anytime when needed.” (IDI, women service user, Beneshangul-Gumuz)*

*Shift in gender norms influencing access-use of RMNCH*. Today, the productive and reproductive roles are shifting. The tasks (such as taking care of children, washing clothes, fetching water, collecting wood, and dismantling small nuts) delineated for women are becoming shared with men and vice versa. The shifts in gender norms are attributed to structural factors, such as the introduction of a country-wide social network system (such as HDA) and livelihood, such as diversification of life-earning jobs.

*“Gender roles are changing to some extent, and women and men are entering into activities that were traditionally categorized as “masculine” and “feminine” activities, respectively. Men are involved in activities such as washing clothes, fetching water, and collecting wood when there is a shortage of human labor in the household…Further, constructing and dismantling small huts is shifting from female to male as people are beginning to live in permanent places.”*

The shifts in gender norms caused negative and positive shifts in access to RMNCH. Women becoming over-occupied by roles previously played at home by men (such as looking after cattle and collecting wood) deterred the uptake of RMNCH services. The changing trend of sociocultural preferences and perceptions regarding who should provide RMNCH/FP services affected access use in DRS. For example, traditionally, people preferred female health workers for birth attendance in Benishangul-Gumuz, which is now altered.

### 3.3 Intersectionality of gender, sociocultural, structural, and programmatic factors in RMNCH

As indicated in Fig 2, the intersectionality of gender, a web of interaction across structural, sociocultural, and programmatic factors intersects gender in determining access to and uptake of RMNCH/FP services. The multilevel bi-directional relations and intersectionality of gender are demonstrated in the following examples. 1) Men and religious leaders are socioculturally assigned to safeguard FP service utilization. 2) RMNCH policies impacted the sociocultural engagement of religious leaders in fighting harmful traditional practices/HTPs (such as rape, early marriage, and biting) and programmatic arrangements (such as the recruitment of HEWs who are entirely female). 3) Sociocultural preference for female health professionals for providing RMNCH services leads to recruiting females or convincing the community to accept RMNCH services provided by male health professionals.

*“In our community, if a woman is raped and goes to a clan leader for arbitration, the perpetrator is made to pay compensations in the form of cows, goats, or camels as punishment…. Arbitrations for such cases are made in a traditional way or in the court of law.” (Religious leader, Berhale District)*

*“…There is no such kind of sex preference so far; however, to provide services as client preference would be difficult because we do have a shortage of female midwifery).**”** (Health worker, Berhale District)*

#### 3.3.1. Intersectionality of structural conditions, gender and RMNCH

Structural contexts/conditions (policies/laws, empowerment opportunities, participation in leadership, and control-over economy and resources) intersected with gender in influencing access to and use of RMNCH in DRS, as discussed below.

*Laws and policies*, *gender*, *and RMNCH*. Although legal frameworks addressing gender norm challenges and inequalities in RMNCH are in place, knowledge and implementation of the laws among respondents, including health workers, are limited. Community and religious leaders adopted laws on the harmful traditional practices of HTPs for use in local sociocultural contexts.

*“If a husband beats his wife, the punishment is 180 Birr, but if he causes head injury, he will provide her with broth made from goats through ‘Erro’ and pay the 180 Birr. In the case of rape, the punishment will be 12 cows, and he will be free.” (WCY Affairs Office, Gulina District)*

Existing policies suggest that RMNCH service delivery be modeled in a gender-sensitive manner that addresses programmatic-gender intersectional barriers to RMNCH uptake. For example, addressing the issue of female birth attendants preferred by the sociocultural context of DRS is one example of a poorly implemented gender policy in the health system. Health systems/facilities have attempted to recruit adequate female health workers/midwives and persuade the community to accept male health workers. Accordingly, the intersectionality of laws with gender, culture, and programs influenced the uptake of RMNCH services in the DRS.

*“…Afari men do not want their wives to be seen by male health professionals during pregnancy or childbirth. As a result of which they prefer to be assisted by their mothers, family members or TBAs.” (Clan leader, Afar region)*

Some regions resolved the issues of female birth attendants through joint efforts of health professionals, communities, and religious leaders.

*“….now, there is not much challenge regarding preference for female birth attendants although it still existed. And, providing services entirely according to clients’ preference would be difficult because we have a shortage of female health professionals (midwifery).**”** (KII, Health worker, Berhale District)*

*“…We are working with religious leaders….Whatever activity we do, the community would not accept us unless it is endorsed by the religious and community leaders (KII, Health worker, Somali)*

*Control-over resources and decision making*, *gender*, *and RMNCH*. The power to control resources intersects with gender norms in influencing access to RMNCH. Most of the time, men were empowered to control resources, such as selling livestock and occasionally jointly deciding.

*“Either the man or the woman can sell the animals. The husband and the wife jointly make the decision. But in those households where the man is dominant, he can sell the animals and have the money for himself.” (KII, religious leader, Bambasi District)*

Across the DRS, while men do not usually interfere with or exert much influence on other RMNCH services (immunization, antenatal care, and postnatal services), family planning is less favored by men.

*“Regarding decision making on the use of RMNCH services: women alone can decide, but in case of family planning, she must discuss with her husband for approval.” (KII, WCA, Lare District in Gambela)*

Access, use, follow-up, and discontinuation of RMNCH/FP services relied on men’s approval of the services and financial support according to their sociocultural and religious privileges.

*“I have experienced lots of Muslim women hiding from their husbands to use FP service because they hadn’t got an agreement from their partner. Sometimes they discontinue follow-up schedules because of financial problems. Other than FP services, women can decide to seek services; this is actually with an intention that males don’t oppose the rest of the services like ANC, PNC, child vaccination.” (KII, District Health office, Dangur District)*

*Participation in leadership and RMNCH*. Across the DRS, participants mentioned that men held important leadership positions, such as administering customary and religious institutions, kebeles, meetings, and events. Hence, men’s leadership checks matter against religious dogmas or customs, irrespective of the women’s knowledge and demand for RMNCH/FP services. Women have fewer positions in such positions but largely participate in rituals such as births and genital cuttings. This revealed the intersectionality of the leadership structure and gender norms, as described in.

*“In Muslim community (like Berta community), women are not allowed to hold the important position because of ‘sharia’….” (KII, community leader, Assosa District)*

*“Men hold the position of a religious leader, clan leader, kebele administrator…women also take leadership in birth rituals and FGM practices.” (KII, religious leader, Hamero in Somali)*

Nonetheless, there has recently been a shift in such trends in which girls/women hold important positions (e.g., women’s network/HDA leadership) in a government structure.

*“Previously, women do not hold any leadership positions. But this is showing improvement, and they are assuming positions in different leadership positions. They are now more observed in government positions and associations organized by non-governmental organizations.” (Clan leader, Telalak District)*

*Empowerment and education opportunities*, *gender disparity*, *and RMNCH*. Gender disparity was observed when accessing information on RMNCH/FP services. Women have access to RMNCH/FP information and educational opportunities. Most of the time, the health extension program (HEP) uses different educational strategies such as home visits, HDA schemes, trained TBAs, and traditional means of exchanging information (the ‘*Dhagu System’* in the case of Afar region) to empower and access the women with RMNCH information. By contrast, although men have better access to general information than women, their access to RMNCH information is minimal because they are excluded from RMNCH/FP educational strategies. Moreover, mobility in pastoralist communities has predominantly prevented men from educational opportunities that interact with their restrictions on women’s FP uptake.

*“…Married women and those who have children have better knowledge of RMNCH/FP issues. This is because of the focus on strategies involving women in the programs like the HEP, and there are some audio-visual education sessions in waiting areas of HCs. Besides, women get information on RMNCH through the HDA, and community representatives, including a mother whose child is treated and cured, will participate as part of the mobilization team to teach the community and promote health…” (NGO representative in Beneshangul-Gumuz)*

*“…RMNCH/FP services and education provided in the nearby health facility do not reach fathers and husbands or encourage their participation in RMNCH/FP care. Because the current HEP targets mothers and children while fathers and husbands seem to be less focused for RMNCH/FP services…unless they get such information from other sources.” (Women FGD, Hamero in Somali)*

*“… Pastoralists, who move from place to place in search of livestock feed and water, have less knowledge i.e., about service availability or its benefit…. As a result, I believe, their awareness is less to support RMNCH/FP utilization…” (KII, LSA, Awubere district)*

#### 3.3.2. Programmatic factors intersecting gender on RMNCH uptake

The facilities and systems by which RMNCH services are delivered determine access use. The unavailability or punctuality of health professionals, respectfulness, perceived lack of skills and capacity, distance at which facilities are located, transport and ambulance services, lack of essential supplies and equipment, friendly services, and gender sensitivity of the services are some of the factors affecting RMNCH access use.

*“…as a result of women experiencing that Implanon removal service is provided by appointment and not by their immediate request because of lack of provider. It inhibits their long-acting FP service seeking. As a result, they prefer depo as it is a one spot service with no requirement for removal…” (HEW, Afar)*

*“…If a woman comes bleeding or delivered at a health center and there is no water facility for washing, food to eat…she would think she is inviting death to herself and may not come again. The problem with water shortage in the HC is serious, and it affects us not to come to the HC.” (women, FGD, Gambella)*

The availability of health workers, perceived competencies, and user-friendliness of the RMNCH service intersected with gender norms. Sociocultural beliefs and religious dogmas interacted and determined how the RMNCH/FP program and services needed to be arranged. For example, 1) the absence of female health professionals (even in the presence of males) determines RMNCH uptake as females socioculturally prefer to attend births, and 2) youth-friendly RMNCH services seem unthinkable in Muslim dominated settings across the DRS due to the religious dogmas.

*“In our culture, a man is not allowed to see a woman’s body. Most men in our community believe that male health professionals should not assist their wives’ delivery at a health facility. This is the main reason why women in the community do not have institutional delivery…sometimes, when it deems necessary to deliver at health facility traditional birth attendants will accompany the women” (Clan leader, Afar)*

*“…We have a shortage of health care staff. There is a high turnover of staffs as they leave to other regions. During the last political turmoil, it could be said that most health facilities were almost closed. More than 450 staffs left, especially those who initially came from highland regions [non-Somalis]…” (KII, Somali)*

*“…There is no adolescent’s reproductive health officer down at district or kebele level. This situation has hampered utilization of RMNCH / FP services by young people… we are trying to make adolescent and youth RH services accessible for all…but the key challenge would be that it is socio-religiously forbidden to address unmarried youths on RMNCH/FP” (KII, Benishanzul)*

*“…We as young people do not have access to FP services. One thing it is a taboo to engage in sex before marriage. It also is not expected from young ladies to be sexually active and get pregnant before marriage. So, access to FP at health facilities is generally poor,even if youngs want to you [Male, FGD, Afar region]*

*“…young people may access FP or other services such as abortion from private facilities by hiding themselves from the public.[Female, FGD, Gambella]*

#### 3.3.3. Intersectionality of sociocultural factors and gender and RMNCH

In this study, the sociocultural factors intersecting gender in influencing RMNCH embrace sociocultural practices and socio-demographic/economic, and socio-psychological dimensions. Different drivers contributing to intersectional gender and sociocultural influences in RMNCH service uptakes were illustrated by drawing on the lived experiences of the study participants.

*Sociocultural practices and gender*. Their husbands felt responsible for caring for their wives during their pregnancy and delivery. Men arrange transport and other expenses as women seek RMNCH services. Cultural beliefs that supposed parents to care for and support their children influenced the men to cover costs as the women visited health facilities for child vaccination, treatment, and postnatal follow-up services. Nonetheless, sociocultural beliefs did not encourage men to develop a custom of accompanying women on their way to seek RMNCH services other than delivery, although the norm is shifting.

*“The husband has a cultural responsibility to take care of his wife during her pregnancy regarding covering costs and expenses related to delivery and labor. The office has been educating men on the issue. Other than this, it is the woman who, in the majority of the time, takes children for vaccination or follows her antenatal or postnatal follow-up alone. But during delivery or labor, it is culturally mandatory for the husband to attend to her. It is not out of rudeness for the men not to be helpful on the other RMNCH/FP services; however, it originates from their cultural practices.” (WCY Affairs Office, Gulina District)*

*“…Currently, education being given to the community to promote male involvement and support in helping a woman financially, psychologically, and physically. The community only accepts if the man is supporting his wife or accompanying her to a health facility during labor and for delivery services, but support for other services is not common…” {KII, Benishangul Gumuz)*

Some husbands refrain from accompanying their wives during ANC visits because of the fear of HIV testing.

*“…Men do not participate and support women because they have many partners, and they do not allow their wives to undertake HIV testing. They don’t also want to accompany their wives because of fearing to undertake HIV testing…” (HEW, Gog Woreda)*

As a result of their experiences attending deliveries at home, some TBAs scare women with the potential absence of female health professionals from health facilities (hence, males see their private body) and cesarean section as a dangerous delivery mode. They discouraged the utilization of health facilities for delivery services. TBAs engaged in women’s education and empowerment opportunities determine the type of information they disseminate and that influence women’s uptake of RMNCH/FP services. This demonstrates how sociocultural practices interact with empowerment structures and intersect gender norms to influence RMNCH/FP and FP uptake.

*Socio-demographic and economic disparities*, *gender and RMNCH*. The interaction and intersection between gender norms and socio-demographic factors, such as education and rural-urban residence, determined how women and men responded to RMNCH issues and uptake. For example, an unfair gender division of labor increases the burden on women. This limits their potential and time to look for self-development opportunities and education, address other intellectual needs, and attend to their own and their children’s health. Similarly, women who were relatively educated or informed of RMNCH and lived in urban settings had a better capacity to manage their productive and reproductive roles and RMNCH uptake. Access to social support from close relatives, siblings, or even productive offspring facilitates women’s access to health facilities.

*“…We do not have time as we are more burdened with our household activities than men. There is a challenge to go to the health center anytime we want due to the burden we have with our household chores; it is difficult to use the services as needed. However, some women in our community get support from their educated and economically productive children, who may visit health centers anytime they want. They are very advantageous; they have given birth early, and now they are getting this support. Women who get health facility in their vicinity are more advantageous” (FGD female, Telalak District Afar)*

Women in rural areas who have lower formal educational attendance and are overburdened with roles, and men in the pastoralist regions of Afar and Somali, where mobile health services are available, reportedly have lower access to information and knowledge about RMNCH than their counterparts.

*“… Pastoralists, who move from place to place in search of livestock feed and water, have less knowledge i.e. about service availability or its benefit. They give more value to sustaining their livestock than their health. As a result, I believe, their awareness is less…” (KII, LSA, Awubere district)*

*“…Men are not usually present when we provide health education targeting women and children. They usually go away for income-generating activities and some to care for their livestock. During such times, they do not participate and miss the health education sessions…” (KII, Afar region)*

*Intersectionality of religion*, *gender and access-uptake of RMNCH*. Religious leaders have informal power to influence women’s uptake of RMNCH/FP services. In Muslim-dominated settings, men are responsible for safeguarding the religious doctrine, whereas women don’t defend their husbands’ sayings. Religious leaders/husbands recited verses from the Quran to defend their position when the women presented with FP demand, resulting in unmet FP needs in the DRS.

*“…At one time in the past*, *several women refused to seek RMNCH/ FP services*. *Then religious leaders held a strong debates in the presence of the women for and against the use of FP service*. *Eventually*, *those who produced supportive evidence from the Quran have triumphed*. *(KII*, Bambasi District in Benishangul-Gumuz*)*

Religious and clan leaders in most settings of the DRS discourage FP service use, especially the insertion of foreign materials like IUD and Implanon into the body: they condemned the act as “sin.” The debate among religious leaders regarding the appropriateness of FP across the DRS has not yet been resolved. Some religious leaders wanted to leave their decisions to their partners instead of imposing sanctions.

*“…The community is largely in favor of RMNCH service that does not include FP. Religion is motioned as a pretext for not using RMNCH services. Those against family planning services assert that only Allah can feed its creatures. They claim that no one should get worried about the size of their family*.

*In contrast, there are also other Muslims in favor of FP services and child spacing. Hence, the people who belong to the same religion have divergent opinions on FP services.” (KII, Bambasi District in Benishangul-Gumuz)*

The engagement of religious and community leaders determines gender norms and the use of RMNCH, as evidenced by the successful introduction of the Human Papilloma vaccine in the Somali region.

*“…We are trying to work with religious leaders to introduce new vaccines or campaigns. The community would not accept us in whatever activity we do unless the religious and community leaders endorse it. For example, last time we introduced a vaccine called HPV [Human Papilloma Virus: which prevents cervical cancer] ….We finally used community and religious leaders to convince the communities…I can say that almost 100 percent of the Mosques in Somali region have been transmitting positive messages about the vaccine during Friday prayers…” (KII, Somali)*

*Intersectionality of socio-psychological factors and gender*. This section describes the perceptions of the DRS intersecting gender pertaining to RMNCH. Perceptions in the community regarding existing RMNCH/FP inhibited the use of these services. Participants often negatively evaluated FP services due to misperceived side effects (e.g., FP causes infertility), religious sanctions attached to it (especially among Muslim-dominated regions of Afar and Somali), and values for children and fertility were deep-embedded in the cultures and religions. Men, particularly those in rural settings, often prefer large families. This shaped men to inhibit their wives from using FP.

*“… Using injectable family planning causes swelling of the body on some women and excessive bleeding during menstruation on other women. I tried it one or two times, but it didn’t cause me anything…” (TBD)*

*“…Women fear FP services’ side effects; they often think FP may bring infertility. On the other hand, there is a misunderstanding that TT vaccination may make labor difficult while giving birth…” (HEW, Gog District)*

A belief that says “it is improper to show women’s private body to a man other than a husband” refrained women from delivering at health facilities, mainly where they are by male health professionals. Men also do not want male healthcare professionals to provide antenatal care. Antenatal and delivery services provided by male health professionals are undermined and not seen as services.

“*We also recommend recruiting female midwifery to assist our women during the birth*. *Our women would not volunteer to be assisted by a male midwife unless they experience severe labor pain*. *Even in our Islam religion*, *“even for a dead female*, *the process of postmortem care would be done by women only” ((KII*, *a religious leader in Telalak District in Afar)*

*“…Afari men do not want their wives to be seen by male health professionals during pregnancy or childbirth*. *As a result of which they prefer to be assisted by their mothers*, *family members or TBAs*.” (Clan leader, Afar region).

## 4. Discussion

This study has identified six key findings. First, consistent with studies from LMICs, RMNCH/FP service uptakes were influenced by gender norms and structural, sociocultural, and programmatic underlying conditions [11–15,42,45,46]. Intersectionality theory postulates the non-additivity of the effects of sex and other social stratifiers (such as religion and ethnicity, among others) but extendibility to other domains (such as policies, distribution of power over money and resources, institutional practices, settings where people are born and grow, etc.) for reciprocal interaction across multilevel influences on health, including RMNCH [47–51].

Second, this study found that intersections of sociocultural, religious, and leadership positions made men influential but engaged less in the RMNCH/FP program. Although women were interested in RMNCH/FP uptake, men were less supportive and were not involved in ongoing RMNCH interventions. Even if they engage, they favor other maternal health interventions such as ANC, delivery, PNC, and child health services. FP was less supported by RMNCH services than ANC, child immunization, and delivery (and hence is illustrated in detail). Evidence indicates that CPR is the lowest at 3% in the Somali region [41], which is far less than the expected contraception rate of 55% in 2025 [52]. FP utilization and unmet demand were influenced by multilevel sociocultural, structural, and programmatic intersectionality of gender. Most of the proposed RMNCH interventions exclude men. For instance, although women want to use FP services, men who dominate leadership positions and are structurally responsible for safeguarding religious dogmas and controlling finances condemn the use of FP services because of their poor participation in the RMNCH.

Similarly, a study from Kenya reported that women’s inability to make decisions influences FP uptake [53]. Furthermore, religion is also one of the social factors that influence FP services as studies from Muslim-dominated territories reported little controversy on RMNCH issues other than FP [54–57]. A study from Kenya reported controversy regarding the religious permissibility of FP [53]. Studies from Muslim-dominated African, Asian, and European territories indicated tranquility (presented by the *Quran*, *Shariah*, *Sunnah*, and culture) as an essential factor of contraception. Tranquility is an important purpose of a Muslim family, and is achieved through marriage. Procreation is not the exclusive purpose of sexual relations in a Muslim family; it should support and endorse tranquility rather than disrupt it. Thus, contraception helps families achieve tranquility by having children when they want them and are prepared to have them.

The little opposition to FP has two grounds: a belief that it is infanticide, which is condemned in *Quran*; and if FP has become a government policy, it would be against the religion’s tradition as it prevents greater power. FP is permitted when there are observed and expected health risk occasions for mothers or pregnancy life that are against a family’s tranquility [54–56]. Therefore, the extent to which the sociocultural/religious intersectionality of gender influenced FP uptake is determined by the dominance of men in religious affairs, and the permissibility option prevailed in that particular setting.

Third, this study found that structural conditions (political empowerment, resource control, and leadership) and interventions influenced RMNCH/FP utilization by building on sociocultural factors that intersect with gender. For instance, consistent with other studies, mutual support in women’s networks such as the WDA and leadership reduced financial problems in seeking RMNCH/FP services [58,59]. Women’s engagement and participation in structural conditions in the form of WDA loses the financial inability of women and decisions that are closely associated with the lack of control over resources, and hence improved access to and uptake of RMNCH/FP services.

Fourth, in addition to structural rearrangements, the women’s psychosocial empowerment interventions by the Ethiopian Health Extension Program (HEP) that used WDA and TBAs in DRS have increased women’s knowledge and demand for RMNCH/FP (although some TBAs were negatively influenced). Women are more positively influenced by RMNCH/FP messages from HEP and community-based structures [52,60]. For instance, WDAs frequently met with HEWs, supported fellow women, and facilitated education and linkages to RMNCH services [47,48,52], and women’s education increased contraceptive rates in Ethiopia [41,43,52,54]. Studies indicate that family level empowerment in countries such as Iran and WDAs (wherein TBAs are included) in Ethiopia resulted in supportive beliefs and better antenatal, delivery, and contraception service utilization rates [54,58,59,61].

Fifth, programmatic factors also intersect with gender and sociocultural, and structural conditions that influence RMNCH/FP. For example, women need a socioculturally and psychologically accommodating environment and culturally acceptable and competent female healthcare workers. Although women are becoming tolerant (owing to better access to education and engagement in the program), men are still unconvinced about the provision of RMNCH/FP services by male healthcare workers. The recruitment of competent female healthcare providers at grassroots health facilities would boost the appropriateness and user-friendliness of RMNCH/FP services. The men felt at ease to let their women actively become involved in WDAs and have contact with HEWs and other health workers at facilities or community-based mettings, which in the overall advanced women’s access, knowledge, and uptake of RMNCH/FP services. Ethiopia’s HSTP-II health sector transformation plan and global strategies also highlight the need to ensure high-quality health services tailored to address sociocultural barriers [52,54–57]. However, the inaccessibility of user-friendly RMNCH/FP largely affected RMNCH/FP service uptake, particularly among those seeking long-acting FP and delivery services (as most frontline (HEWs who are females) deployed at grassroots levels are not well equipped with the skills of providing these services). Gender norms indicated that some shared roles by men and women concerning RMNCH services, such as ANC and child immunization and deliveries (men emotionally and financially supportive), were often associated with perceived benefits, preference for the sex of birth attendants, and cultural practices of traditional birth assistance by TBAs.

Sixth, cross-intersectionality (reciprocal and multilevel) of gender norms was present in RMNCH service uptake in the DRS. Formal laws shaped sociocultural-religious practices and RMNCH programs (and vice versa) and intersected gender. The 2005 penal code of Ethiopia and the revised family code of July 2000 criminalized HTPs, such as rape, FGM, early marriage, and gender-based violence. There were also gender-responsive policies and regulations that monitored, trained, and assigned human resources and engaged sociocultural and religious leaders in reducing HTPs [62,63]. Nonetheless, evidence indicates that restrictive, socio-religiously debated laws are not effective for some RMNCH services, such as FP [54,55]. In some regions where empowerment schemes involving men and religious leaders in programmatic arrangements were limited, the recruitment of male RMNCH/FP providers (especially birth attendants) remained challenging, because men HPs were not culturally appropriate for observing the sexual organs of female clients. Hence, support programs by policies may not be a significant factor in RMNCH uptake without considering social-cultural and gender initiatives. Ethiopian HSTP-II attributed the challenges of addressing gender disparities in health to the limited enforcement of existing laws/policies on the rights of women and girls and capacity among health care workers to design and implement gender-responsive health services [52,60]. This finding aligns with reports on gender-health systems [14,15,41,44].

The role of gender in general and women’s increased participation in sociocultural and political governance structures, particularly in Ethiopia, is rapidly changing because of the policy environment, education of women, women taking part in income-generating activities, and urbanization, which has promoted the role of women in delivering health services both among HEW and the development army health education personnel. These interactions produced significant intersectional gender influences on RMNCH perceptions, access, and uptake in Ethiopia’s DRS.

### 4.1 Limitation of the study

This study has some limitations. The fact that the interviewers were predominantly male (only one female) could have biased the responses of the female FGD/KII participants. Although the study covered a large geographic setting and a significant number of districts and villages, further representation of the sociocultural and religious contexts and variations across the DRS would better present further gender intersectionality and maternal health research in those regions.

## 5. Conclusions

Overall, the current state of RMNCH/FP service access and utilization is affected by multiple social and programmatic factors that intersect with gender. Women who were overburdened with household chores were less likely to use RMNCH/FP services. Structural, sociocultural, and programmatic conditions and their interaction with each other and their web of intersectionality with gender determined access to RMNCH/FP. Men’s dominance in control-over resources and decisions and sociocultural andreligious affairs cross-interacted and intersected reproductive gender norms and set barriers to the uptake of RMNCH/FP. Socio-religious leadership that predominantly engaged men’s lack of gender culturally competent or adaptive RMNCH programs, and disproportionate gender-based participation in the RMNCH empowerment scheme negatively influenced the move toward access to RMNCH. FP service utilization was the most negatively affected program across developing regions compared to other RMNCH services, mainly because of sociocultural-religious perspectives safeguarded by men (husbands and religious leaders), who were stratitegically marginalized to participate in RMNCH/FP.

Promising structural efforts and opportunities, such as access to RMNCH education, improving access to health care, women’s self-help networks, and participation in community-based leadership of women’s health leadership, would remain an important intervention in addressing the influence of gender intersectionality on RMNCH. Strengthening the shift in gender norms by reducing women’s work burden, increasing their participation in decision-making and resource control, and improving their knowledge and perceptions would address women’s needs, gender norms, and intersectional gender influences on RMNCH uptake, particularly FP. Women empowerment initiatives, such as women’s networks, self-support, and access to RMNCH/FP education, should be strengthened and complemented by men’s engagement and sociocultural and religious leaders. A systemic understanding of multilevel (structural, sociocultural, and programmatic) cross-intersectionality of gender inequalities should inform gender-responsive RMNCH strategies that empower communities, engage sociocultural and religious leadership, and integrate programs in developing regions of Ethiopia.

## Supporting information

S1 File Interview guides. (RAR)

S2 File Transcribed data. (RAR)
